# Incidence of Non-alcoholic Fatty Liver Disease (NAFLD)/Non-alcoholic Steatohepatitis (NASH) in the Female Population of North Karnataka: A Cross-Sectional Study

**DOI:** 10.7759/cureus.66257

**Published:** 2024-08-06

**Authors:** Geeta S Desai, Santosh Hajare, Sandesha Ghorpade

**Affiliations:** 1 Department of Gastroenterology and Hepatology, Karnatak Lingayat Education Society's (KLES) Dr. Prabhakar Kore Hospital and Medical Research Centre, KLE Academy of Higher Education and Research, Jawaharlal Nehru Medical College, Belagavi, IND

**Keywords:** bmi, liver fibrosis, fibro scan, diabetes, nafld

## Abstract

Background: India is currently facing an epidemic of type 2 diabetes mellitus (T2DM) and obesity which are high-risk factors for the development of non-alcoholic fatty liver disease (NAFLD). A non-invasive tool, the vibration-controlled transient elastography (VCTE; FibroScan, Echosens, Paris, France) is used to diagnose NAFLD.

Aim: To identify the prevalence, spectrum, and metabolic determinants of NAFLD in Indian adult women using liver function tests (LFT) and non-invasive FibroScan (liver stiffness measure, i.e., LSM score) of the liver through a cross-sectional population-based study in the city of Belagavi.

Methods: The subjects were screened for the presence of liver disease with a detailed history, anthropometric measurements, LFTs, blood sugars, and FibroScan of the liver to assess liver steatosis and liver fibrosis.

Results: The study included 2448 women with 860 (35.13%) having NAFLD (controlled attenuation parameter {CAP}≥275 dB/m) as detected by FibroScan. Nearly, 58.8% of the participants with T2DM had NAFLD. Participants with NAFLD had higher BMI and waist circumference. When univariate logistic regression was applied, those with T2DM were 14.5 times (95% CI, 4.55, 6.52) likely to have CAP≥275 dB/m. Similarly, those with higher BMI>23 mg/m^2^ were 1.34 (95% CI, 1.68, 2.37) times more likely to have CAP ≥275 dB/m. The risk of NAFLD increases by ~1% for every one-year increase in age.

Conclusion: NAFLD in women is the most common non‑communicable disease in India; a prevalence of 35.13% was observed in the present study in women. Higher BMI, presence of metabolic risk factors, and incremental age were associated with a high risk of developing NAFLD in women.

## Introduction

Indian National Association for Study of the Liver (INASL) describes non-alcoholic fatty liver disease (NAFLD) as a spectrum ranging from non-alcoholic fatty liver (NAFL), non-alcoholic steatohepatitis (NASH), NASH with fibrosis, NASH-cirrhosis, and NASH-hepatocellular carcinoma (HCC) [1]. In NAFLD, ˃5-10% of hepatocytes are fatty which is not associated with other known causes of steatosis like alcohol, viruses, drugs, toxins, autoimmune disease, or iron overload but frequently accompanied by Insulin Resistance (IR) [2]. Recent data suggests NAFLD is a hepatic component of metabolic syndrome [3]. Therefore, these subjects also have comorbidities like type 2 diabetes mellitus (T2DM), and hypertension apart from obesity (cardiovascular risk factors) leading to high cardiovascular, all-cause, and liver-related mortality [3-5].

High rates of NAFLD in adults >18 years are being observed in the Middle East and South America, followed by Asia, North America, Europe, and Africa [3]. India is currently facing the brunt of T2DM and obesity while NAFLD has catapulted into a substantial public health problem in India [6]. A meta-analysis published in India reported a NAFLD prevalence of 38.6% [1,7], and up to 20% of those who are obese may develop NASH [5]. Nearly, 30-40% of individuals have elevated liver enzymes at diagnosis [5]. The gender-specific NAFLD prevalence was 39.4% among males and 35.4% among females in India [7]. 

Patients with NAFLD/NASH and significant fibrosis are mostly asymptomatic thereby early-stage diagnosis and management play a significant role in prevention and treatment [8]. Non-invasive diagnostic tools can now be used to assess the likelihood of significant fibrosis and predict the risk of disease progression and decompensation [9]. One of the frequently used non-invasive tools is vibration-controlled transient elastography (VCTE; FibroScan, Echosens, Paris, France) across the country. It uses the ultrasonic attenuation of echoes (controlled attenuation parameter, CAP) which supplements the ability to quantify hepatic steatosis. The results are fairly accurate since the CAP values gradually increase, with increasing severity of steatosis. The European Association for the Study of the Liver (EASL) has recently recommended a CAP cut-off of ≥275 dB/m for the detection of hepatic steatosis [1,10]. The present prospective community-based cross-sectional study, aimed to identify the prevalence, spectrum, and metabolic determinants of NAFLD in Indian adult women using liver function tests (LFT) and non-invasive FibroScan (liver stiffness measure, i.e., LSM score) of the liver.

## Materials and methods

Study design

This cross-sectional descriptive survey was carried out among women at the Department of Gastroenterology, Karnatak Lingayat Education Society's (KLES) Dr. Prabhakar Kore Hospital and Medical Research Centre, Jawaharlal Nehru Medical College, Belagavi, Karnataka. Women aged 18 to 80 without a prior history of liver diseases were included in the study. They were screened for liver disease with detailed history, anthropometric measurements, liver function tests (LFTs), blood sugars, and FibroScan of the liver to assess liver steatosis and liver fibrosis. The study excluded women who were consuming a significant amount of alcohol or who had overt cirrhosis, previously diagnosed hepatitis B virus or hepatitis C virus infection, biliary diseases, and pregnant females.

Study procedure

A written consent was obtained from all the women before enrollment. The screening was done by recording medical history. Anthropometric measurements included height (cm), weight (kg), waist circumference (cm), hip circumference (cm), and waist-to-hip ratio. BMI as per the Asian BMI criteria and blood pressure were recorded. After an overnight fast, a FibroScan of the liver and biochemical tests (serum glutamate pyruvate transaminase (SGPT) and serum glutamic-oxaloacetic transaminase (SGOT), were performed. Subjects were categorized as having NAFLD if there was evidence of fatty liver on imaging. Subjects with abnormal LSM on FibroScan with or without abnormal liver function tests were labelled as NASH.

All the participants eligible for the study underwent a FibroScan test. This is a non-invasive method that has been proposed for the assessment of liver fibrosis by measuring liver stiffness. It uses the principle of transient elastography. Vibrations of mild amplitude and low frequency (50 Hz) are transmitted by the ultrasound transducer probe, mounted on the axis of a vibrator, which induces a plastic shear wave that propagates through the underlying tissues. The degree of hepatic fibrosis can be determined by the liver's hardness. The results were represented in kilopascals (kPa). The test takes about 10 minutes. The eligible participants were instructed to lie supine on the examination table. On the right side of the lower chest wall (6th or 7th intercostal space), a technician positioned the FibroScan (FibroScan Mini 430, Echosens, Paris, France; Device ID-03662264001192; August 18, 2021) probe between the ribs. The liver was then subjected to a given a series of painless pulses. A total score for LSM was produced after the data were recorded on the apparatus. A gastroenterologist used this data to predict the possibility of fibrosis or fatty liver. FibroScan measures the LSM score in kPa and the CAP score in decibels per meter (dB/m) [3,11].

A CAP score can be between 100 and 400 dB/m, steatosis with CAP is graded as follows: (S), ≤ S0 ≥ S1, ≥ S2, and ≥ S3 were <275 dB/m no steatosis, > 275 dB/m, > 319 dB/m, and > 337 dB/m, respectively [12]. Liver fibrosis has four stages: F0 to F1 fibrosis score (no or little liver damage; 2 to 7 kPa), F2 fibrosis (moderate liver scarring; 7.5 to 10 kPa), F3 fibrosis (severe liver scarring; 10 to 14 kPa), and F4 fibrosis (cirrhosis; 14 kPa or higher) [3].

Ethical committee review

All procedures were followed according to the guidelines of the Indian Council of Medical Research (ICMR), to protect the rights of the patients. Approval for the study has been obtained from the KAHER (KLE Academy of Higher Education and Research) Ethics Committee on Human Subjects (KAHER/EC/21-22/014).

Statistical analysis

Data was analyzed using IBM SPSS Statistics for Windows, Version 21 (IBM Corp., Armonk, NY) and Microsoft Excel, Version 2013 (Microsoft Corporation, Redmond, WA). Categorical variables are given in the form of a frequency table. The chi-square test analyses categorical variables. The Shapiro-Wilk test and QQ plot check the normality of the variable. Mann-Whitney U test compares the distributions of variables over FibroScan; a p-value less than or equal to 0.05 indicates statistical significance.

## Results

**Figure 1:**
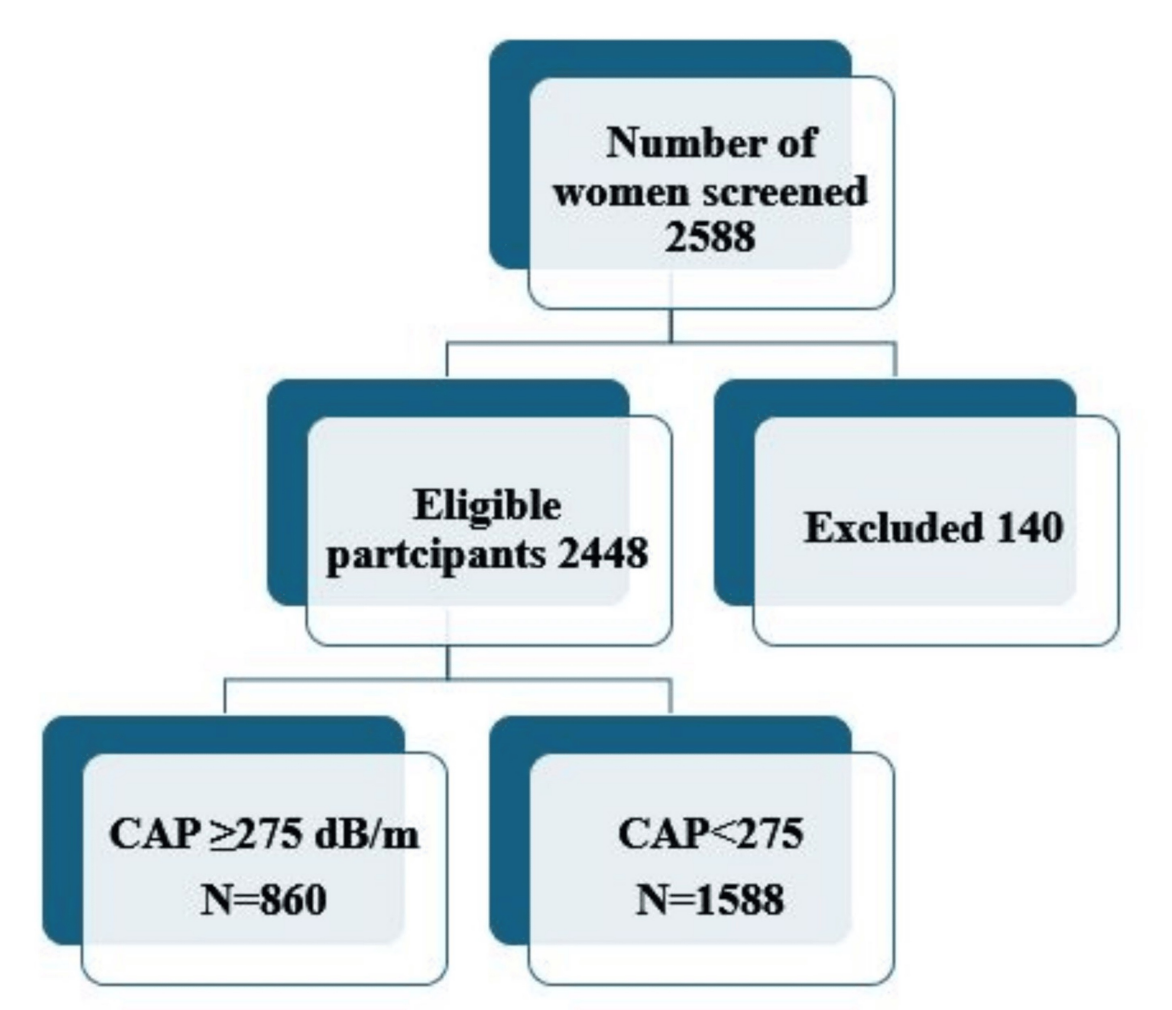
Patient disposition. CAP: controlled attenuation parameter, dB/m: decibels per meter, N: number of participants in the study.

A total of 2588 healthy women were screened; 140 participants did not meet the inclusion criteria therefore, the total cohort comprised of 2448 women (Figure 1). The baseline demographics of all the selected participants are shown in Table 1.

**Table 1:** Baseline demographics (N=2448). WC: waist circumference; T2DM: type 2 diabetes mellitus; HT: hypertension; SGOT: serum glutamic-oxaloacetic transaminase; SGPT: serum glutamic pyruvic transaminase; CAP: controlled attenuation parameter; dB/m: decibels per meter; FS: fibrosis score kilopascals (kPa); NASH: non-alcoholic steatohepatitis; NACH: non-alcoholic cirrhosis. Alcohol within permissible limits: 10 mg/day; BMI (India): underweight <18.5 kg/m2, normal weight 18.5–22.9 kg/m2, overweight >23 kg/m2, obese >25kg/m2.

Variable	Groups	Number (%)
Age (years)	Age<20	17 (0.69)
	Age:20-35	639 (26.1)
	Age:35-50	1174 (47.96)
	Age:50-65	539 (22.02)
	Age>65	79 (3.23)
BMI (mg/m^2^)	Underweight	105 (4.29)
	Normal	476 (19.44)
	Overweight	438 (17.89)
	Obese	1429 (58.37)
WC (cm)	<80	221 (9.03)
	>80	2227 (90.97)
T2DM	Yes	923 (37.7)
	No	1525 (62.3)
HT	Yes	747 (30.51)
	No	1701 (69.49)
HT and DM together	Yes	567 (23.16)
	No	1881 (76.84)
SGOT (U/L)	Normal	1300 (53.1)
	Elevated	1148 (46.9)
SGPT (U/L)	Normal	1717 (70.14)
	Elevated	731 (29.86)
CAP score (dB/m)	Normal	1588 (64.87)
	S1	384 (15.69)
	S2	63 (2.57)
	S3	413 (16.87)
FS (kPa)	F0-F1	1475 (60.33)
	F2	589 (24.09)
	F3	270 (11.04)
	F4	111 (4.54)
NASH	No	1797 (73.41)
	Yes	651 (26.59)
NACH	No	2351 (96.04)
	Yes	97 (3.96)

The participants included in the study were screened for fatty liver by ultrasound examination. Further, they underwent FibroScan evaluation for steatosis (CAP in dB/m) and fibrosis (LSM in kPa). The mean (±SD) age of the participants was 42.94 (±11.4) years and 1174 (47.96%) women were in the age group of 35-50 years (Table 1) while 1813 (74.06%) women were aged below 50 years. The mean BMI of the participants was 26.04 (±4.6) mg/m2 with 1429 (58.37%) of the participants categorized as obese. The mean waist circumference (WC) of the participants was 98.11 (±17.3) cm with 2227 (90.97%) of the participants having WC>80 cm. The mean liver enzyme value for SGOT was 47.03 (±32.31) U/L while for SGPT, it was 48.5 (±32.83) U/L. The mean CAP score of the participants was 263.69 (±53.5) dB/m. The personal history of the participants revealed that 923 (37.7 %) had T2DM while 747 (30.51 %) had hypertension. Table 2 depicts the summary of all the variables.

**Table 2:** Summary of all demographic variables. WC: waist circumference; SGOT: serum glutamic-oxaloacetic transaminase; SGPT: serum glutamic pyruvic transaminase; CAP: controlled attenuation parameter; dB/m: decibels per meter; FS: Fibrosis score kilopascals (kPa), SD: standard deviation.

Variable	N	Mean	SD	Median	Min	Max
Age (years)	2448	42.94	11.42	42	18	83
BMI (mg/m^2^)	2448	26.04	4.61	26	2.6	41.5
SGOT (U/L)	2448	47.03	32.31	38	8	182
SGPT (U/L)	2448	48.5	32.83	38	6.2	211
FS (kPa)	2448	7.23	3.54	6.2	2.7	50.5
CAP score (dB/m)	2448	263.69	53.5	253	165	391
WC (cm)	2448	98.11	17.3	94	43	156

**Figure 2:**
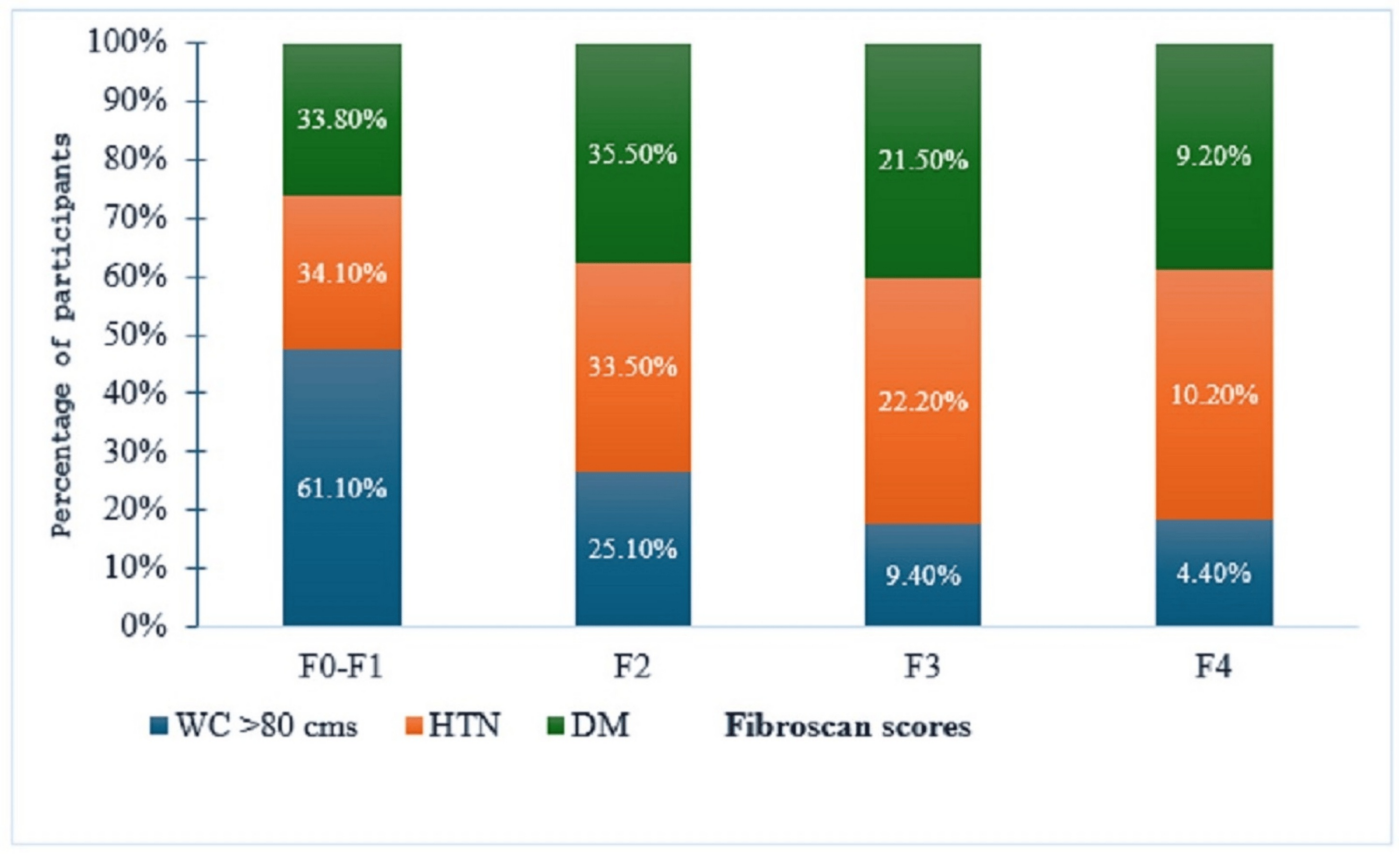
FibroScan scores associated with comorbidities (N=2448). WC: waist circumference; DM: diabetes mellitus; HT: hypertension.

**Figure 3:**
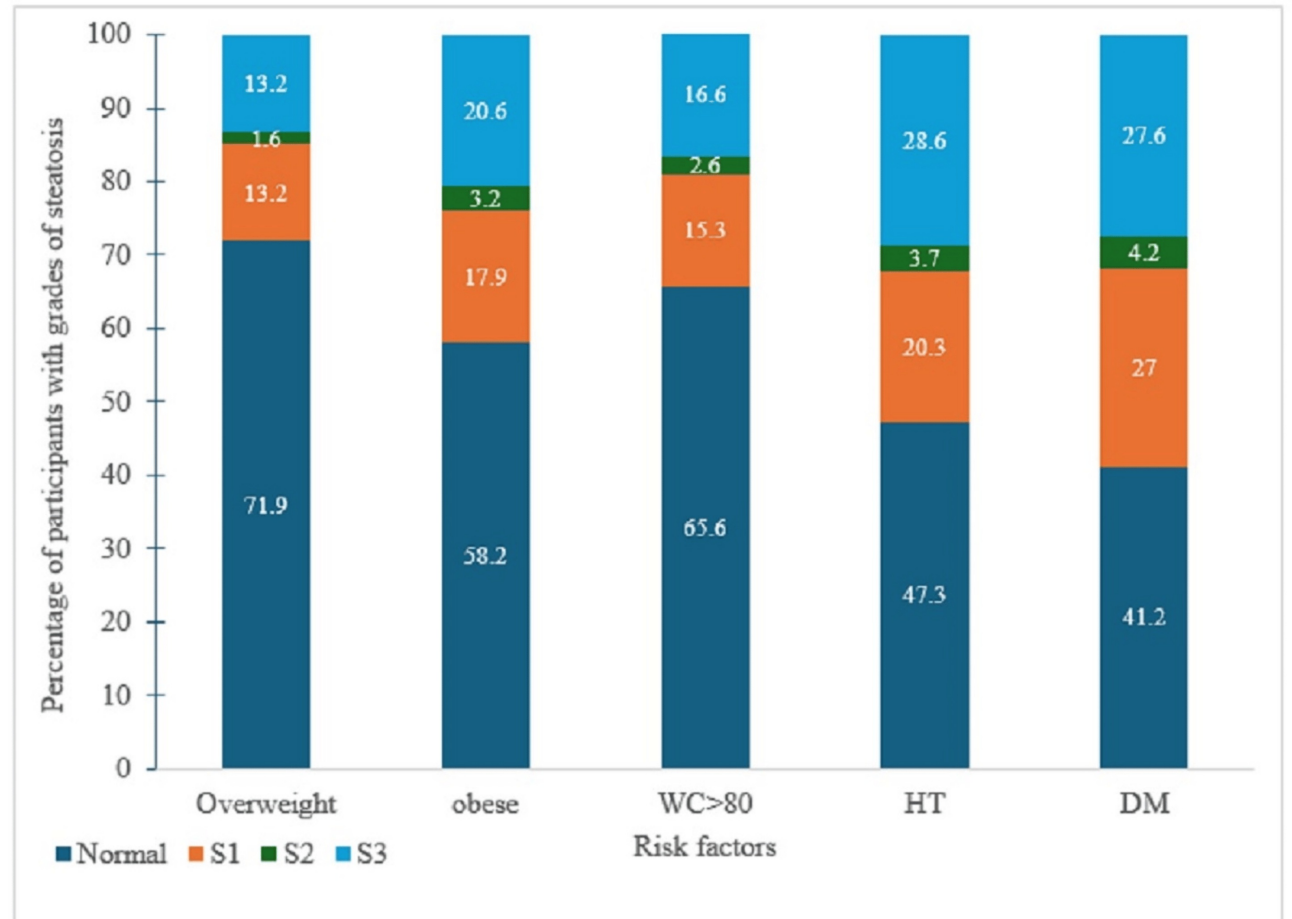
Steatosis scores associated with comorbidities (N=2448). WC: waist circumference; DM: diabetes mellitus; HT: hypertension.

FibroScan score 2 (F2) was observed in 328 (35.5%) of the participants with T2DM, 250 (33.5%) of the participants with hypertension, and 558 (25.1%) of the participants with WC >80 cm (Figure 2). A greater number of participants with both comorbidities (DM+HT) had higher FibroScan scores (p<0.001). A greater number of the participants with T2DM had steatosis of varying grades (Figure 3).

The study results noted that 35.13% of the participants had NAFLD (CAP≥275 dB/m) as detected by FibroScan®, 15.69% of participants had steatosis grade 1 (S1), 2.57 % of participants had steatosis grade 2 (S2), and 16.87% participants had steatosis grade 3 (S3) (Table 1). Not surprisingly, 35.13 % of the participants had NASH and 4.40% of the participants had non-alcoholic cirrhosis. The proportion of participants with NAFLD was upscaled to 62% in the presence of BMI>23 kg/m2, T2DM, and hypertension together. Apart from higher BMI, participants with NAFLD had elevated liver enzymes, WC>80 cm, and high Fibroscan scores (Table 3).

**Table 3:** CAP scores as per demographic variables and associated risk factors. WC: waist circumference; T2DM: type 2 diabetes mellitus; HT: hypertension; CAP: controlled attenuation parameter. High CAP score ≥275 dB/m; low CAP score <275; p-value ≤0.05 is statistically significant.

Variable	Low CAP	High CAP	P-value
	(n %)	(n %)	
BMI			
Underweight	81 (77.1)	24 (22.9)	<0.001
Normal	360 (75.6)	116 (24.4)	
Overweight	315 (71.9)	123 (28.1)	
obese	832 (58.2)	597 (41.8)	
WC			
WC<80 cm	128 (57.9)	93 (42.1)	0.028
WC>80 cm	1460 (65.6)	767 (34.4)	
T2DM	380 (41.2)	543 (58.8)	<0.001
HT	353 (47.3)	394 (52.7)	<0.001

The mean age of participants with NAFLD was 45.15 years and the mean age of controls (low CAP <275) was 41.7 years. The mean BMI of participants with NAFLD was 27.3 mg/m2 while that of controls was 25.3 mg/m2. The mean SGOT in participants with NAFLD was 54.7 U/L while that of controls was 42.8 U/L. The mean SGPT in participants with NAFLD was 59.4 U/L while that of controls was 42.6 U/L. This indicates higher levels of liver transaminases in NAFLD. A greater number of participants with T2DM had NAFLD (58.8%).

Table 4 depicts the results of univariate and multivariate logistic regression for NAFLD. When univariate logistic regression was applied to calculate the odds ratio and its 95% CI for each of the independent variables, those with T2DM were 14.5 times (95% CI, 4.55, 6.52) likely to have CAP≥275 dB/m. Similarly, those with higher BMI >23 mg/m2 were 1.34 (95% CI, 1.68, 2.37) times more likely to have CAP ≥275 dB/m. The risk of NAFLD increases by ~1% for every one-year increase in age.

**Table 4:** Results of univariate and multivariate logistic regression for NAFLD. T2DM: type 2 diabetes mellitus; HT: hypertension, OR: odds ratio, NAFLD: non-alcoholic fatty liver disease. P-value ≤0.05 is statistically significant.

Risk factors	Unadjusted. OR (95%CI)	Adjusted. OR(95%CI)	P-value
Age (Year)	1.03 (1.02,1.03)	1.01 (1.01,1.02)	< 0.001
Obese	2 (1.68,2.37)	1.68 (1.39,2.03)	< 0.001
T2DM	5.45 (4.55,6.52)	4.59 (3.73,5.66)	< 0.001
HT	2.96 (2.47,3.54)	1.2 (0.97,1.5)	0.099

## Discussion

This cross-sectional study found that 860 (35.13%) out of 2448 women had NAFLD diagnosed using FibroScan in Belgavi (India). This is more likely to be just the tip of the iceberg since the adoption of less healthy and more Westernized lifestyles is directly proportional to the rising prevalence of NAFLD all around the globe. This ultimately signals higher morbidity and mortality in the coming years. A meta-analysis comprising 23,581 participants noted a gender-specific NAFLD prevalence of 35.4% (95% CI 23.5- 48.3%; I^2^ 98.5%) among adult women [7]. In a study in Bhopal (India), the prevalence of NAFLD was 43.6% [3]. Community-based studies reflect the prevalence rate of NAFLD in urban Mumbai as 16.6%, urban Chennai as 32%, and rural Haryana as 30.7% across gender [13-15].

A meta-analysis demonstrated a higher overall prevalence of NAFLD among adults in South Asia, especially in those with metabolic disorders. In the South Asian population, NAFLD was associated with T2DM, hypertension, dyslipidemia, general obesity, central obesity, and metabolic syndrome [4].

Along similar lines, the present study also showed a higher prevalence of NAFLD in women with T2DM and obesity. Women with larger waist circumference were considerably more likely to develop NAFLD. This parameter has been well-established in past research too as strong associations with NAFLD.

The waist circumference (WC) is an anthropometric measurement of obesity related to abdominal (central visceral) adiposity, which is an important predictor of the development of T2DM and NAFLD [16]. Asians are more vulnerable to T2DM and have a higher prevalence of NAFLD, which may be caused by an abnormal deposition of visceral fat [3].

All causes of death, including cardiovascular disease, myocardial infarction, and ischemic stroke, increase across all age groups in the order of no NAFLD, grade 1 NAFLD, and grade 2 NAFLD, and with age [17]. The incidence rate of cardiovascular disease for patients with T2DM and no NAFLD in the 20-29-year age group was 0.40 per 1000-person years; whereas, for patients with T2DM with grade 2 NAFLD in the ≥70-year age group, the rate was 23.25 per 1000 person-years [17]. Therefore, in the present study women with T2DM and NAFLD are at high risk of future CVD. The most important risk factor for NAFLD is T2Dm which is incidentally also a clinical predictor of adverse outcomes like developing clinical decompensation and hepatocellular carcinoma [18].

This calls for action to early screening in overweight and obese women. FibroScan (transient elastography) is an easy, rapid, accurate, and non-invasive screening test that measures liver stiffness through the estimation of the velocity of propagation of a shear wave through liver tissue. LSM carried in high-risk patients can funnel down the number of patients who require biopsy evaluation [19]. There are various studies substantiating the use of FibroScan in NAFLD. In a previously reported study from the same center, the sensitivity of the FibroScan score prediction with an area under the curve (AUC) of 0.80 was found to be 74.6% and the specificity was 74.3% [20]. Wong et al. reported the AUC values of transient elastography for F3 or higher and F4 disease as 0.93 and 0.95, respectively [21]. Another study from India showed similar efficacy of FibroScan in NAFLD in patients from Northern India [19].

Limitations

This is a cross-sectional study data. Though the pooled cohort was large, the lipid profile and duration of metabolic disorders were not available. It is a single-center community study thereby giving only a glimpse of the prevalence of NAFLD in women.

## Conclusions

This is one of the largest cross-sectional single hospital-based studies focused on screening for non-alcoholic fatty liver disease (NAFLD), particularly among women. NAFLD in women is the most common non‑communicable disease in India; a prevalence of 860/2448 (35.13%) was observed in the present study in women. Higher BMI, presence of metabolic risk factors, and incremental age were associated with a high risk of developing NAFLD in women. Community-based screening or mass screening is needed to arrest this epidemic through the implementation of awareness programs and healthy lifestyle practices. Early diagnosis is the key which can be achieved with easy, reliable, and non-invasive tools like FibroScan.
